# To: Patient functionality and walking speed after discharge from the intensive care unit

**DOI:** 10.5935/0103-507X.20210021

**Published:** 2021

**Authors:** Kelly Cattelan Bonorino, Roberta Rodolfo Mazzali Biscaro

**Affiliations:** 1 Adult Intensive Care Unit, Hospital Universitário Polydoro de Ernani de São Thiago, Universidade Federal de Santa Catarina - Florianópolis (SC), Brazil.

**TO THE EDITOR**

The subject approached by Silva and Santos’ work ‘Patient functionality and walking speed after discharge from the intensive care unit’^(1)^ is highly important, considering the need for the functional and physical capabilities assessment in patients after discharge from an intensive care unit (ICU); this is aimed to provide quality indicators to guide the clinical practice.^(2-4)^ However, caution is necessary when analyzing and interpreting their results.

Aiming to measure the patients’ functional ability after discharge from the ICU and before hospital discharge, 10 meters walking test was performed. However, this test is not appropriate in the hospital and critically ill patient settings and represents an important study bias. Additionally, it is reported that each patient underwent the test three times at each assessment time (discharge from the ICU and just before hospital discharge), and the calculated three measurements mean was considered for analysis purposes. We ask why carrying out three tests and which methodology was the study based on.

Likely a learning effect may have occurred in the first test,^(5)^ which could underestimate the distance and speed, and consequently reduce the mean speed.

The sample characterization in table 1 displays a heterogeneous sample (postoperative of myocardial revascularization surgery patients, acute myocardial infarction patients, among others), and this may have influenced the results. Additionally, unstable angina was described in six patients, likely implying submaximal functional capacity tests. Also, in these cases, these tests would be formally contraindicated.^(4)^ Besides, no mention was made of safety measures for these tests (interruption criteria, post-test care).

Additionally, the authors report a significant improvement during the hospitalization (p < 0.001). On the post-ICU test, the mean speed was 0.48m/s, and before the hospital discharge, it was increased to 0.71m/s.

We ask how much this difference was (0.23m/s) relevant for important clinical outcomes, and how much should one conclude that this difference (although statistically significant) would reflect in significant clinical status and functional capacity changes. Therefore, we suggest not extrapolating the results to an improved functional capacity or functionality - i.e., the interpretability (the degree to which qualitative significance can be attributed to the assessed quantitative score) and the minimal clinically important difference, in which the difference in a test result should be necessary for significant changes in clinical status.^(4)^

We stress that the application of an assessment tool should be based on its practicality and clinical relevance. Clinimetric properties knowledge is decisive to select an assessment tool, which should have good clinical applicability and present previously demonstrated robust measurement properties.^(4)^
